# Childhood ADHD, Going Beyond the Brain: A Meta-Analysis on Peripheral Physiological Markers of the Heart and the Gut

**DOI:** 10.3389/fendo.2022.738065

**Published:** 2022-03-01

**Authors:** Ameanté Payen, Michelle J. Chen, T. Grace Carter, Ryan P. Kilmer, Jeanette M. Bennett

**Affiliations:** ^1^ Health Psychology PhD Program, University of North Carolina at Charlotte, Charlotte, NC, United States; ^2^ Department of Psychological Science, University of North Carolina at Charlotte, Charlotte, NC, United States

**Keywords:** ADHD (attention deficit and hyperactivity disorder), gut micobiota, childhood, adolescence, heart rate reactivity, meta-analysis, biopsychosocial model of health and disease, complex and adaptive systems

## Abstract

Attention-Deficit/Hyperactivity Disorder (ADHD) is the most common neurodevelopmental disorder diagnosed in children. Questions regarding its increased diagnostic rates and pharmacological treatments in developing children have led to a more holistic review of the multi-system pathophysiology observed in ADHD. The dopaminergic neurotransmitter system, known for its influence on reward-motivated behaviors and motor control, and the frontostriatal systems, that mediate motor, cognition, and behavior, are associated with ADHD’s development. However, studies have shown that these neural systems do not wholly account for ADHD’s multilayered and heterogeneous symptom presentation. For instance, the literature suggests that emotional dysregulation, the inability to regulate one’s emotional responses to provoking stimuli, is associated with increased risk for social impairment in ADHD. A broader examination of physiological systems in children with ADHD has found potential markers in the heart-brain and gut-brain axes that correspond with certain behaviors associated with emotional dysregulation in recent studies. Hence, the purpose of this meta-analysis is to aggregate ten applicable published case studies and analyze task-related heart rate reactivity (HRR; n = 5 studies) and gut microbiota (n = 5 studies) data in children with and without ADHD. Data from a total of 531 youth with ADHD and 603 youth without ADHD revealed significant small and medium effect sizes for higher Chao1 levels and Actinobacteria levels in the ADHD group, respectively, but no evidence of altered task-related HRR. Thus, further research into multi-system psychophysiological measures of emotional dysregulation and ADHD is warranted. The clinical, empirical, and educational implications of these findings are discussed.

**Systematic Review Registration:**

https://www.crd.york.ac.uk/prospero/, identifier PROSPERO (CRD42021236819).

## 1 Introduction

Attention-Deficit/Hyperactivity Disorder (ADHD) is one of the most commonly diagnosed childhood neurodevelopmental disorders (1), with an estimated lifetime prevalence rate of 9.4% in the United States (2) and 7.2% worldwide (1, 3). Further, there has been a 42% increase since the early 2000s in ADHD diagnoses among children and adolescents in the U.S. (2). This increase is of particular concern given ADHD’s association with cumulative socioemotional, educational, and financial repercussions throughout the lifespan (1). For example, youth with ADHD are more likely than their neurotypical counterparts to experience adverse symptoms and outcomes such as lower academic attainment, impaired psychosocial functioning (4), increased substance use (5), and reduced occupational success in adulthood (6). With this growing prevalence, direct health care expenditures and associated indirect costs have also increased (7); the resulting annual societal costs (e.g., medical care, caregiver strain, education, reduced work productivity) accompanying childhood ADHD in the U.S. are estimated to be $124.5 billion (8). Furthermore, one of the primary expenses for families is the annual incremental costs of prescription medications, which were about $2,200 more for families of children with ADHD than children without ADHD (7).

Current hypotheses concerning the etiology and maintenance of ADHD, in addition to genetic factors, propose underlying metabolic and neuropsychological pathology, such as omnigenic (e.g., the perinatal environment) and environmental (e.g., socioemotional development, diet) factors. The cardiovascular system has also been indicated in regulating and mediating the autonomic stress system and the brain (1). This study aims to review recent evidence for the cardiovascular and gastrointestinal microbiome underpinnings of emotional dysregulation in children with ADHD, discuss implications and provide recommendations for treatment and future research.

### 1.1 Diagnosis of ADHD: Current Considerations

Recommendations for the diagnostic evaluation of ADHD include a comprehensive interview addressing the child or adolescent’s developmental, medical, and psychosocial history, including academic performance, peer relations, and family functioning (9). Other components of the evaluation regularly include symptom rating scales (e.g., Brief Rating Inventory of Executive Function [BRIEF]; 10, 11), neuropsychological testing (e.g., intelligence testing, continuous performance tasks), and a thorough physical exam to augment information collected during the clinical interview (9). Primary sources of information for younger children are parents, teachers, or other caregivers, with older children and adolescents more involved in the evaluation (12). Moreover, structured and semi-structured diagnostic interviews, including the Diagnostic Interview Schedule for Children (DISC-IV; 13), are often used to assess ADHD symptomatology, as well as the presence of comorbid psychiatric diagnoses that share similar symptoms, such as difficulty concentrating and impulsivity (e.g., Oppositional Defiant Disorder [ODD], Conduct Disorder [CD], and General Anxiety Disorders; 14). Additionally, ADHD diagnoses are often classified by one of three broad subtypes: Inattentive, Hyperactive-Impulsive, and Combination, with broad behavioral diversity within and across the categories (1).

Still, there are significant issues associated with current diagnostic processes. Comprehensive evaluations for ADHD can be time-intensive and costly, often requiring upwards of five to eight hours of testing and thousands of dollars (15). A primary care physician may diagnose to circumvent these difficulties; however, they often lack the time and expertise to differentiate ADHD from other conditions (16). Additionally, the general agreement among different informants (e.g., parents, teachers, other caregivers; 17) in different settings (e.g., school versus home; 18) has also been shown to range from fair to moderate (19, 20). Further complexity is introduced into the diagnostic process by the influence of multiple sociocultural factors that can hinder diagnostic test specificity and sensitivity and, more broadly, impact differential diagnosis. For instance, early sociocultural influences such as adverse childhood experiences and poverty have been associated with disruptive behaviors and ADHD symptoms (21). Research indicates that ethnicity may predict ADHD diagnosis and treatment type, even after controlling for socioeconomic status (22).

Such findings regarding the discrepancies in diagnostic and treatment rates of ADHD have highlighted concerns surrounding the clinical process, including the appropriateness of intelligence testing and its application among non-European/Caucasian individuals (23), and the high false-positive/negative rates of continuous performance tasks (24–26). Given the implications of the diagnosis for intervention planning, the need to substantiate ADHD diagnoses with objective clinical indicators, such as reliable physiological markers, is crucial. Furthermore, physiological measures can provide another means by which clinicians, researchers, and educators can measure treatment efficacy and develop tailored treatments to address individual physiological needs.

### 1.2 Treatment of ADHD: Current Considerations

Historically, ADHD has been treated with potent psychostimulants such as methylphenidate or amphetamine (27, 28). While these drugs effectively manage symptoms for many youth, they are legally categorized as controlled substances and have a high likelihood of abuse (27, 29). In addition, there is limited knowledge regarding adverse side effects and long-term effects on the developing brain (30). Thus, non-stimulant medications such as antidepressants and CNS anti-hypertensives have been utilized to assist in ADHD symptom management (27–32). Unfortunately, pharmacotherapy lacks precision; neurotransmitter levels are universally altered throughout the whole brain rather than within specific, targeted regions (e.g., 33). However, observations regarding the impact of such medications have led researchers to hypothesize insufficient or reduced dopamine and norepinephrine levels as mechanisms supporting the development and maintenance of behavioral and cognitive symptoms associated with ADHD.

Psychotherapy is frequently implemented in conjunction with medication. Behavioral therapy treatments often help children navigate peer relationships, which can be impaired by difficulties with emotional self-control (34). Organizational skills training can also be integrated into treatment to target executive functioning deficits commonly exhibited by youth with ADHD (35). However, the individualistic scope and often expensive nature of psychotherapy sessions (34) frequently fail to manage the evolving and concurrent biopsychosocial influences on the developing child. In sum, neither pharmacotherapy nor psychotherapy can adequately address the causes or symptoms of ADHD. Therefore, it is necessary to continue developing a broader and more holistic view of the pathogenesis of ADHD to improve diagnosis, treatment, and quality of life for children with the disorder.

### 1.3 The Human as a Complex and Adaptive System

**Figure 1:**
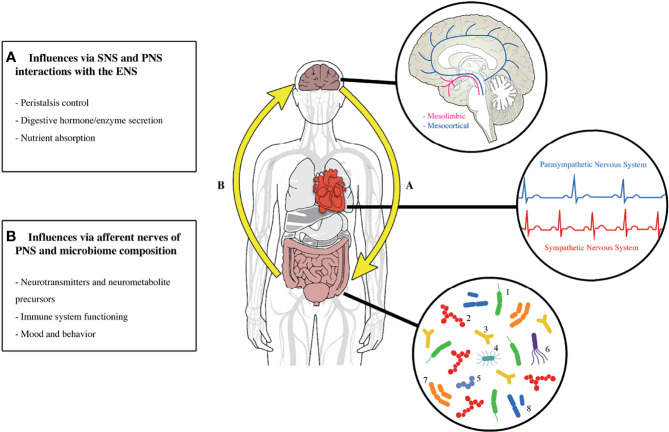
A depiction of how the whole human affects overall functioning and behavior with an emphasis on the gut-brain axis and heart. The gut, heart, and brain operate as interdependent units. **(A)** Highlights the top-down communication; the brain alters heart and gut functioning *via* the autonomic nervous system and neuroendocrine hormones. **(B)** Demonstrates the bottom-up communication; gut microbiota directly and indirectly influence neural functioning by producing neurotransmitters and precursor metabolites as well as modulating metabolic and immune pathways. Gut-brain communication is estimated to be ~20% top-down (efferent) and ~80% bottom-up (afferent), leading some to identify the gut and enteric nervous system as a sensory system that alters cognition, perception, mood, and behavior. The *brain pop-out* illustrates 2 of the major dopamine neuropathways: mesolimbic responsible for motivation and reward and mesocortical responsible for attention. The *heart pop-out* demonstrates the general influence of the SNS and PNS on heart rate. The *gut pop-out* provides the common and rare microbiota that typically colonize the human gut. *Microbiota Key:* 1 = Bacteroides, 2= Faecalibacterium, 3 = Bifidobacterium, 4 = Enterobacteriaceae, 5 = Streptococcus, 6 = Escherichia coli, 7 = Eubacterium, 8= Clostridium. SNS, sympathetic nervous system; PNS, parasympathetic nervous system; ENS, enteric nervous system. Graphic by Shawn James.

The current biomedical approach frames ADHD as a neurocognitive disorder driven primarily by dysregulated dopamine and norepinephrine levels in the brain (36, 37). This explanation oversimplifies the complexity of the human body and the series of interconnected systems that produce behavior, as illustrated in **Figure 1** (38, 39). The brain prepares, plans, and instructs autonomic and voluntary activity to create complex actions such as words or movement, suggesting that top-down physiological pathways are critical factors in ADHD (40). That said, brain functioning is influenced by both external stimuli *via* the sensory systems and internal stimuli within the body *via* afferent autonomic nervous system activity and neuroendocrine pathways (41, 42). Thus, the dynamic integration between these bottom-up peripheral communication pathways and the cortical system is critical to the homeostatic modulation of the brain’s output, thoughts and behaviors (38, 39).

#### 1.3.1 Emotional Dysregulation in ADHD

Though not considered an official criterion of a DSM-5 diagnosis, the high comorbidity between emotional impulsivity and ADHD is well documented (43–45). Emotional regulation refers to an individual’s (in)ability to appraise and respond to stimuli during an emotional or stressful state adaptively and beneficially (44). Despite the propensity of children with ADHD to display emotionally impulsive behaviors, the physiological evidence for emotional dysregulation in ADHD has not been consistently documented (46). Nevertheless, neurological evidence supports a shared disruption in the prefrontal cortex and the autonomic nervous system (ANS) in children with ADHD or other emotional dysregulation disorders (44).

Furthermore, recent studies suggest that a deficiency in emotional self-regulation is up to 70% inheritable due to a combination of shared genes and environmental factors (44). Emotional dysregulation is a developmentally stable transdiagnostic component of many disorders that are highly comorbid with ADHD, such as CD and ODD (45, 47). Yet, examining the physiological evidence of emotional dysregulation in ADHD has been challenging due, in part, to the heterogeneous developmental trajectory of ADHD, the multiple body systems involved in emotion regulation, and the non-standardized stress studies (46). Nonetheless, the potential to moderate ADHD symptoms by targeting the physiological structures involved in stress reactivity and emotional self-control may be crucial to understanding and managing the pathophysiology of ADHD.

#### 1.3.2 The Role of the Stress Systems

One of the etiological and developmental underpinnings of ADHD appears to be a function of arousal systems dysregulation (48). Specifically, more recent ADHD models conceptualize the disorder as an emotional *and* neurocognitive disorder, thus expanding ADHD to include the arousal and stress systems: the ANS and the hypothalamic-pituitary-adrenal (HPA) axis (1). Stress reactivity (i.e., the immediate response to a perceived stressor) and emotion regulation are intrinsically linked, and can be examined by measuring peripheral cardiovascular and blood or salivary indicators of neuroendocrine system functioning at baseline (i.e., a non-stressed state) or across a stressful lab-based task (46).

Abnormal diurnal cortisol patterns and acute cortisol reactivity indicating altered HPA axis functioning have also been associated with many adverse behavioral health problems, including impulsivity (49). For instance, children with ADHD were found to have lower cortisol levels than children without ADHD 30 minutes after awakening in the morning and displayed a blunted stress response to a real-life dental visit stressor, in addition to an increase in reported anxiety (50). However, though these studies suggest a possible relationship between altered diurnal cortisol among children with ADHD, a recent meta-analysis of 12 studies found no relationship between a childhood ADHD diagnosis and alterations in lab-based acute cortisol reactivity (46). Together, the phenotypic heterogeneity of the ADHD subtypes and the developmental nature of ADHD raises substantive challenges for physiological ADHD research and requires a whole-body multi-system approach to examine the intersections of underlying behavioral and biological mechanisms.

#### 1.3.3 Evidence for Heart Reactivity Disruption in ADHD

Given the arousal system’s effect on the heart and stimulant medication’s alteration of ANS activity (40), researchers have sought to assess the role of cardiac functioning, specifically cardiac vagal control (CVC), as a proxy for autonomic functioning. As such, heart rate variability (HRV), a measure of the beat-to-beat variations across successive heartbeats, estimates the flexible balance between the parasympathetic and sympathetic nervous systems; in addition, some vagally-mediated HRV measures are linked to mood and emotion regulation (51). Besides HRV, heart rate reactivity (HRR) is a commonly used measure of physiological arousal and stress reactivity (28). Unlike HRV, which measures the inter-heartbeat variations, HRR measures the average beats per minute of the heart after an autonomically arousing event, such as a stressor or a task (40). Both HRV and HRR can provide insight into the sympathetic and parasympathetic nervous systems (14).

In a recent literature review of 35 studies assessing cardiac functioning in children and adults with ADHD, 20 studies examining resting heart rate provided inconclusive results: six reported lower resting heart rate levels, two reported higher resting heart rate levels, and the remainder found no group differences between people with ADHD and those without ADHD (40). However, this weak association may be due to the broad age range included in the review, given that developmental research has found heart rate levels to decrease with increasing age (40). Bellato et al. (40) suggested that future studies examine stress-related changes in heart rate (i.e., HRR), as this approach may be a more appropriate indicator of ANS reactivity and socio-emotional processing.

#### 1.3.4 Evidence for Gut Microbiota Disruptions in ADHD

Another emerging line of ADHD research involves the gut-brain axis, defined as the bidirectional relationship between the gut microbiome and CNS through neural, hormonal, and immunological pathways (52). The gut microbiome is thought to play a role in facilitating the development of neurodevelopmental disorders (53, 54). Microbiota influences the brain and behavior *via* the gut’s ability to synthesize neurochemicals and their precursors (55, 56). Specifically, precursors of monoamines implicated in ADHD (i.e., dopamine and norepinephrine) are thought to be produced by specific gut microbiota (57, 58). These precursors (e.g., phenylalanine and tyrosine) are absorbed through the intestines and enter the body’s vascular system, eventually crossing the blood-brain barrier and potentially influencing monoamine synthesis (56). Differences in the abundance or activity of gut microbiota are posited to correlate with increased symptoms of ADHD (52, 59).

Though research in this area is limited, emerging evidence about the etiologic role of metabolic disruption in ADHD is promising. For example, one early study examining the effects of microbiome differences in neural reward responses demonstrated that an increase in the microbial biosynthesis of phenylalanine was associated with decreased neural reward anticipation, a key characteristic of ADHD (60). Another study found a lowered abundance of *Bifidobacterium* during infancy to be associated with increased risk for ADHD and Asperger’s syndrome-a neurodevelopmental disorder (61). In addition, early exposure to antibiotics and subsequent disruption to the gut microbiota has also been associated with an increased risk of ADHD (62). Furthermore, evidence suggests that nutrition influences ADHD behavior; early malnutrition has been identified as a risk factor for ADHD (63–65).

The lower gastrointestinal tract is home to trillions of microbiota and under the control of the enteric nervous system that is responsible for many regulatory functions, such as regulating the bidirectional communication pathway between the gut and the brain or the “gut-brain axis” (66, 67). Of the six major phyla (i.e., Firmicutes, Bacteroidetes, Proteobacteria, Actinobacteria, Verrucomicrobiota, and Fusobacteria), Firmicutes and Bacteroidetes are the most prevalent in the gut microbiome (68), such that the ratio between these two phyla has been investigated as a potential developmental marker of unbalanced gut microbiota (69). Although the gut microbiota are typically established by age three (68), the Firmicutes/Bacteroidetes concentration ratio has been shown to range from 0.4 (early childhood) to 10.9 (late adulthood; 69). Hence, determining the optimal ecological profile of the health-enhancing microbiota can provide additional treatment avenues and enhance our etiological understanding of ADHD (59, 66, 68).

Although promising, the data on the relationship between ADHD and microbiota are inconclusive. For instance, some studies indicated that people with ADHD have less microbial diversity (70), greater diversity (54), or no difference in bacterial diversity (60) compared to healthy controls. Moreover, several studies found no significant differences in microbiota phyla levels between ADHD patients and controls (54, 70, 71). Still, one study found increased Actinobacteria phylum and decreased Firmicutes phylum in adolescents and adults with ADHD (60). In spite of these varying outcomes, recent clinical trials examining the effect of probiotic treatments in children with ADHD appear to reduce ADHD symptoms (59). However, the literature reviewed for this analysis did not find any published study that quantified the baseline differences in the microbiome between children with and without ADHD. Identifying the specific potential microbiota imbalances is needed to enhance the complementary supplementation with probiotics.

### 1.4 The Present Study

With that backdrop, we examined the evidence for two physiological systems implicated in cognitive and emotional dysregulation: heart rate reactivity and gut microbiota. The following research questions guide this meta-analysis:

1. Is there physiological evidence for emotional dysregulation in the gut or task-induced heart rate reactivity of youth with ADHD?
2. If so, what is the magnitude of the associations between gut microbiome and task-induced heart rate reactivity and ADHD in youth?
3. What are empirical and clinical factors to consider when conducting enteric and cardiac research in youth with ADHD?

## 2 Method

### 2.1 Study Preregistration

Consistent with the *Cochrane Handbook for Systematic Reviews of Interventions’* best practices on research synthesis and meta-analysis, the present study was pre-registered and accepted for transparent reporting on the National Institute for Health Research international register for prospective systematic reviews website, i.e., PROSPERO (CRD42021236819; 72).

### 2.2 Search Strategy and Inclusion Criteria

To assess the evidence of physiological dysfunction in children with ADHD, we conducted a systematic literature search of PubMed (AP), PsycInfo (MJC), ProQuest Dissertations and Theses (TGC), and Web of Knowledge/Web of Science (JMB) databases from January 2021 to April 2021, using the following search terms for gut microbiota studies: (“attention deficit hyperactivity disorder” OR “attention deficit disorder” OR “ADD” OR “ADHD”) AND (child* OR adolescen* OR infant OR pediatric OR youth OR teen*) AND (“gut microbio*” OR “gastrointestinal microbio*” OR “gut flora” OR “dysbiosis” OR “gut brain axis”). The second outcome measure of this meta-analysis was heart rate reactivity. The following search terms were used to identify the relevant studies: (“attention deficit hyperactivity disorder” OR “attention deficit disorder” OR “ADD” OR “ADHD”) AND (child* OR adolescen* OR infant OR pediatric OR youth OR teen*) AND (“heart rate reactivity” or “heart rate responsivity” or “H.R. reactivity” or “H.R. responsivity” or “cardi* reactivity”), consistent with the guidelines of the Preferred Reporting Items for Systematic Reviews and Meta-Analyses (PRISMA; 72). We examined references from retrieved empirical articles and contacted active researchers in the relevant disciplines. We also contacted researchers conducting ongoing clinical trials from publicly available technical reports to acquire additional potentially relevant data.

The criteria for inclusion were studies that (1) used case-control, cohort, or observational designs; (2) examined gut microbiota or measured average heart rate in beats per minute (bpm) during the baseline and task study phases in humans up to 19 years old with an ADHD and non-ADHD control group; (3) included relevant effect size statistics; (4) were written in English; and (5) were published between within the past decade. The exclusion criteria were (1) review articles and (2) randomized clinical trials, primarily due to a lack of a non-ADHD control group. We obtained full-text articles of all studies that met all criteria before extracting the relevant data from each article. Any article for which inclusion status was unclear during the screening process was discussed among reviewers (AP, MJC, TGC, and JMB), and its status was resolved with a consensus decision.

For all studies, ADHD was defined according to the diagnostic criteria set forth by the fourth or fifth edition of the *Diagnostic and Statistical Manual of Mental Disorders* (DSM-IV-TR or DSM-5). For details on the sample, methods, and variables of interest for all studies, please see **Table 1**.

**Table 1:** Summary Characteristics of Papers Included in the Meta-Analysis Separated by Outcome.

Authors, Year	Country	ADHD (n)	Age (years)*	Control (n)	Age (years)*	Outcome	Quality Score
Dekkers et al., 2020 (14)	Netherlands	81	15.0 (1.8)	99	15.1 (1.4)	Heart Rate Reactivity	9
Griffiths et al., 2017 (1)	Australia	229	12.2 (3.0)	244	12.2 (3.0)	Heart Rate Reactivity	9.5
Perrin et al., 2014 (73)	USA	19	10.05 (1.57)	34	9.62 (1.84)	Heart Rate Reactivity	10
Souroulla et al., 2019 (47)	Cyprus	24	10-12	48	10-12	Heart Rate Reactivity	10
Taskiran et al., 2018 (45)	Turkey	48	8.43 (1.68)	22	8.26 (1.5)	Heart Rate Reactivity	10
Aarts et al., 2017 (60)	Netherlands	19^†^	19.5 (2.5)	77^†^	27.1 (14.3)	Gut Microbiota	8
Jiang et al., 2018 (71)	China	51	8.47 (8.47)	32	8.5 (8.47)	Gut Microbiota	10
Prehn-Kristensen et al., 2018 (54)	Germany	14	11.9 (2.5)	17	13.1 (1.7)	Gut Microbiota	9.5
Wan et al., 2020 (74)	China	17	6-12	17	6-12	Gut Microbiota	9.5
Wang et al., 2020 (70)	Taiwan	30	8.4 (1.7)	30	9.3 (2.2)	Gut Microbiota	10

*Age provided either as mean and standard deviation [M (SD)] or as a range depending on how the authors reported it. ^†^These are the sample sizes for the ADHD and control groups utilized in the original study investigating neuro-imaging and gut microbiota. For the present gut microbiota meta-analysis, the sample size from 15 pairs of age-matched ADHD cases and healthy controls was used. ADHD, Attention-Deficit/Hyperactivity Disorder.

#### 2.2.1 Task-Related Heart Rate Reactivity Required Data for Analysis

Consistent with methods used across HRR studies, HRR to laboratory tasks was calculated using the difference in average bpm between the task and baseline phases (28). Sample size, means, *SD*s, and independent *p*-values were utilized to determine the effect size for all included studies. Researchers from one study (i.e., 1) provided additional data upon request to analyze potential ADHD subtype and symptom severity differences. Supplementary data from two studies were used to calculate effect sizes (14, 47).

#### 2.2.2 Gut Microbiota Required Data for Analysis

Gut bacteria composition from fecal samples was analyzed utilizing 16s ribosomal ribonucleic acid (rRNA; 54, 60, 70, 71) and shotgun metagenomic sequencing (74). To obtain the estimated effect sizes, we compared data from at least two studies. All bacterial information, including taxonomic classification and richness and abundance statistics, was reviewed and selected before conducting any statistical analysis to maintain consistency across the studies. Previous studies found evidence of phyla-level differences contributing to metabolic and neurodevelopmental disorders in human and animal studies (75, 76). Thus, the included studies were analyzed at the taxonomic phylum level, which allowed for microbiome comparisons between studies.

For the present analysis, multiple indices were used to analyze the gut microbiota composition across the present analysis. Operational taxonomic units (OTUs) discriminate between microbiota by grouping the number of unique nucleotide sequences within a sample by clusters (54). Alpha diversity, or the number of different types of gut microbiota (59), measured the relative composition of microbiota within the community sample and was represented by Shannon and Simpson diversity and Chao1 indices (71). The means, standard deviation (*SD*), sample size, and *p*-values were used to calculate the effect size. The *SD* and mean were calculated from the supplemental data from one study (60).

### 2.3 Meta-Analytic Approach

Due to the limited number of studies that met the criteria for inclusion (n =5 HRR studies; n=5 gut microbiota studies), bias control and sensitivity analyses could not be conducted. Instead, the two primary analyses included the relevant data from all eligible published gut microbiota and heart rate reactivity studies. Secondary analyses examined associations between specific bacterial phyla concentrations, heart rate reactivity task types, and symptom severity in children with ADHD compared to control groups. The Comprehensive Meta-Analysis (CMA) software was utilized to conduct all meta-analytic statistical tests (Biostat Inc., Englewood, NJ, USA). Microsoft Excel was used to calculate descriptive statistics and create a codebook for the dataset.

## 3 Results

### 3.1 Characteristics of Included Studies

**Figure 2:**
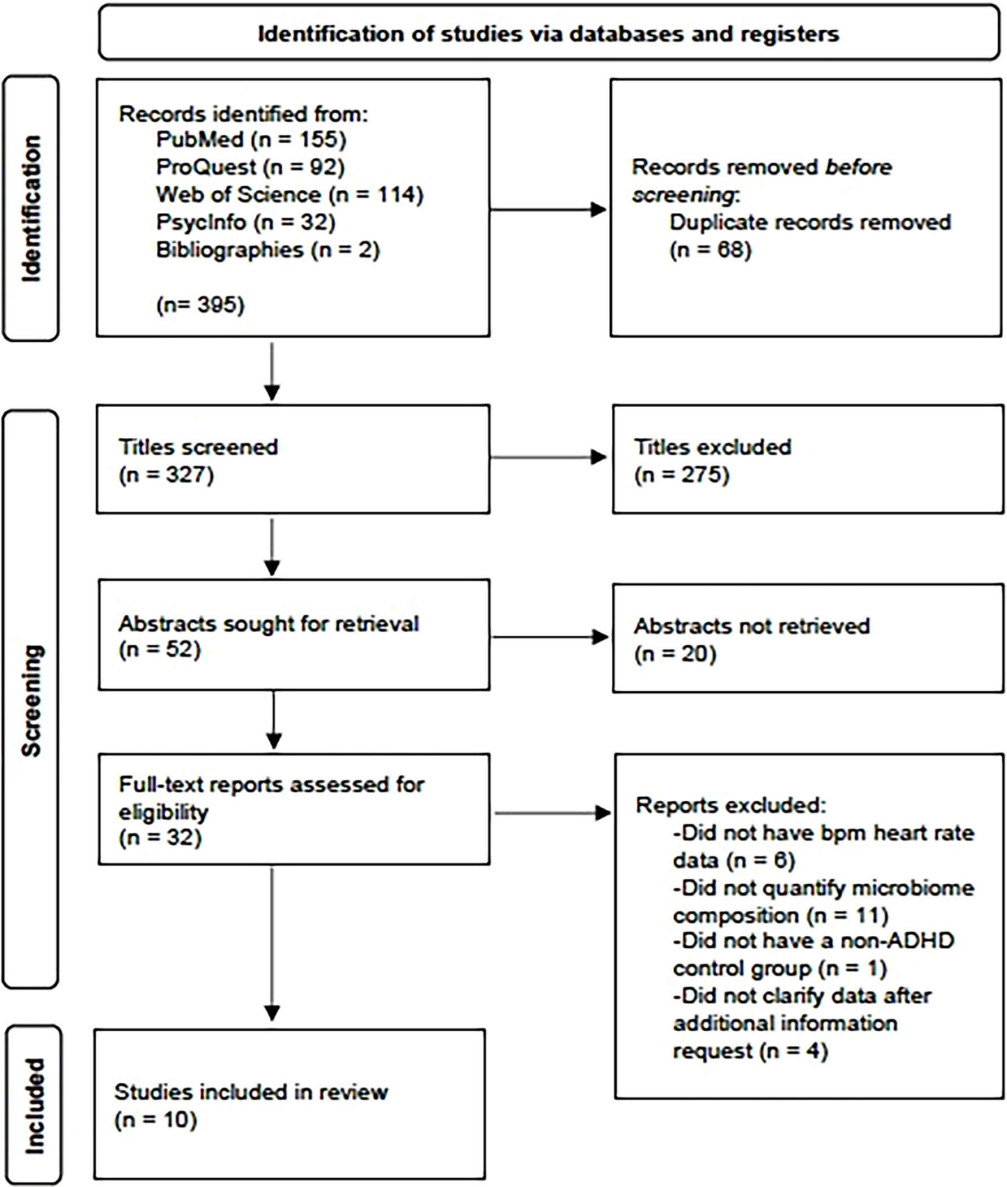
PRISMA flowchart for the number of studies identified, screened, and included in the meta-analysis process. PRISMA, Preferred Reporting Items for Systematic Reviews and Meta-Analyses.

Literature searches yielded 395 potential studies from PubMed (*n*=155), PsycInfo/EBSCO (*n*=32), Web of Science (*n*=144), and ProQuest Dissertations & Theses (*n*=92) as shown in **Figure 2**. Two additional studies were retrieved from the Reference section of included studies. After reviewing titles and abstracts for initial screening for relevant studies, 327 records were excluded. An additional 68 duplicates were removed, and 32 full-text articles were retrieved and individually assessed for eligibility. Of these 32 studies, 10 were included in the present meta-analysis; the remaining articles were ineligible because they did not provide the relevant heart rate data in bpm format (*n*=6), did not provide quantitative data about microbiome composition (*n*=11), did not have a non-ADHD healthy control group (*n*=1), or clarifying data were not provided following data requests for additional information (*n*=4).

All 10 included studies were case-control studies; the five heart rate reactivity studies and five gut microbiota studies that met the criteria for inclusion are outlined in **Table 1**. In total, these studies included 531 youth with ADHD and 603 children and adolescents without ADHD, and, across the ten studies, their ages ranged from 4 to 19 years. Diagnostic methods, medication history, and comorbidities are presented in **Table 2**. Only one study did not report participant medication use (74).

**Table 2:** Summary of ADHD Diagnostic Methods and Relevant Study Factors.

Authors, Year	Diagnostic Methods	Medication History	Comorbidities	Additional Screeners	Inattentive	Hyper-activity	Combined
Aarts et al., 2017 (60)	DSM-IV by trained professionals	Refrained for 48h prior to study	n/a	K-SADS-Dutch	n/a	n/a	n/a
Dekkers et al., 2020 (14)	DSM-IV symptoms from the K-SADS	Refrained from psychostimulants 25h prior to study	7.4% of the ADHD group had comorbid ASD symptoms	SEQ-Dutch; parent/caretaker: DISC-IV-Dutch; parent/caretaker: DBDRS-Dutch; WISC-III-NL-Dutch; WAIS-IV-Dutch	48.1%	2.4%	49.4%
Griffiths et al., 2017 (1)	DSM-IV criteria from referring clinicians	Refrained from psychostimulant 48h prior to study	n/a	CPRS-L	45.85%	n/a	53.71%
Jiang et al., 2018 (71)	DSM-IV criteria by one of two experienced child psychiatrists	Drug-naive	n/a	K-SADS; CPRS	n/a	n/a	n/a
Perrin et al., 2014 (73)	ADD Brown Scales & K-SADS-PL verified ADHD with blind researcher confirmation	Psychotropic medication use was excluded	n/a	CBCL; SDQ; CPRS-48; FAD; SATI; WISC-R	n/a	n/a	n/a
Prehn-Kristensen et al., 2018 (54)	DSM-IV-TR criteria by experienced child/adolescent psychiatrists/psychologists	64.3% refrained for 48h prior to study; 28.6% were drug-naive; 7% received medication prior to study	42.86% of the ADHD group had comorbid ODD	K-SADS-PL; CBCL; FBB-HKS	14.3%	0	85.7%
Souroulla et al., 2019 (47)	Consensus between parent-reported CBCL and child-reported MINI-KID	Psychotropic medication use was excluded	100% of ADHD group met criteria for comorbid ODD or CD	WASI	n/a	n/a	n/a
Taskiran et al., 2018 (45)	ADHD confirmed by a child and adolescent psychiatrist	Drug-naive	47.9% of the ADHD group met the criteria for ODD	CBCL; SDQ; FAD; K-SADS-PL; SATI; CPRS-48	35.4%	8.3%	56.6%
Wan et al., 2020 (74)	DSM-5 by one of two experienced child psychiatrists	n/a	n/a	K-SADS; CPRS	n/a	n/a	n/a
Wang et al., 2020 (70)	DSM-IV-TR criteria by a senior psychologist using K-SADS-E	Drug-naive	n/a	SNAP-IV; WISC-IV; ADHD-RS	n/a	n/a	n/a

Gray highlighted studies report the gut microbiota data. Non-highlighted studies report the task-related heart rate reactivity. ADHD, Attention-Deficit/Hyperactivity Disorder; K-SADS-PL, Schedule for Affective Disorders and Schizophrenia for School-Age Children-Present and Lifetime version;CBCL, Child Behavior Checklist 6-18; SDQ, Strengths and Difficulties Questionnaire; CPRS-48, Conners’ Parent Rating Scale; FAD, McMaster Family Assessment Device; SATI, School-Age Temperament Inventory; WISC-R, Wechsler Intelligence Scale for Children-Revised; ED, Emotional Dysregulation; CD, Conduct Disorder; DBDRS-Dutch, Dutch version of the Disruptive Behavior Checklist; WASI, Wechsler Abbreviated Scale of Intelligence; DISC-IV-Dutch, Diagnostic Interview Schedule for Children; CPRS-L, Conner’s Parent Rating Scale Long Version; DSM-IV-TR, Diagnostic and Statistical Manual of Mental Disorders, Fourth Edition, Text Revision; K-SADS-E, Schedule for Affective Disorders and Schizophrenia for School-Age Children-Epidemiologic Chinese Version; ADHD-RS, Attention Deficit Hyperactivity Rating Scale; FBB-HKS, German ADHD rating scale for children, (Fremdbeurteilungsbogen für hyperkinetische Storungen); n/a, not applicable due to not being reported.

### 3.2 Quality Assessment of Included Studies Methodology

All studies were independently reviewed by at least two authors (AP, MJC, JMB) for quality assessment using a modified Newcastle-Ottawa Quality Assessment Scale to assess cohort and case-control studies (NOS; 77). The NOS rubric is recommended for examining the quality of case-control and cohort clinical research studies (77). Raters assessed potential bias due to selection, comparability, exposure, and reporting categories. The two authors’ scores were then averaged to obtain a single publication-quality assessment score. Only studies with scores of 8 or above out of 10 (n=10), which indicated low or no bias risk, were included in the present meta-analysis (**Table 1**).

### 3.3 Task-Related Heart Rate Reactivity Data

All five studies in the present analysis used an electrocardiogram to measure electrical heart signals (1, 14, 45, 47, 73). The type and duration of tasks are represented in **Table 3**. Baseline heart rate collection ranged from 24 three-second sessions (45) to one continuous five-minute phase (47). Task-related heart rate monitoring lasted from two minutes during the emotional tasks (45, 47) to 13 minutes during a risk-taking virtual peer manipulation task (14).

**Table 3:** Characteristics of the Task-Related Heart Rate Reactivity Assessment in the Included Studies.

Authors, Year	Experimental task type	Duration of Task	Duration of each Resting phase	HRR assessment tool
Dekkers et al., 2020 (14)	Continuous Performance Test	13 minutes	5 minutes	ECG and ICG
Griffiths et al., 2017 (1)	Virtual peer stressor	6 minutes	2 minutes	ECG
Perrin et al., 2014 (73)	Computerized Tower of London planning task	11 minutes	3-7 seconds	ECG
Souroulla et al., 2019 (47)	International Affective Picture System	23 minutes	7 minutes	EKG
Taskiran et al., 2018 (45)	International Affective Picture System	2 minutes	3 seconds	ECG

ECG/EKG, Electrocardiography; ICG, Impedance cardiography.

### 3.4 Microbiota Data

The methods for assessing gut microbiology are shown in **Table 4**. Across the five microbiota studies included in this analysis, the most frequently used technique for identifying, classifying, and quantifying bacteria was 16s rRNA gene amplification sequencing. The 16s rRNA gene is a well-preserved, empirically-supported, highly sensitive ribosomal fragment used to classify and quantify DNA-life forms and identify microbiota (68). Depending on the 16s rRNA gene sequence length, organisms can be identified by species, genera, families, or phyla (68).

**Table 4:** Characteristics of the Microbiota Assessment in the Included Studies.

Authors, Year	Bacteria phyla included in our analyses	Sample	Index	Microbiology Assessment
Aarts et al., 2017 (60)	Actinobacteria, Bacteroidetes, Firmicutes	Fecal	Shannon, Chao1	16s rRNA
Jiang et al., 2018 (71)	n/a	Fecal	Shannon, Simpson, Chao1	16s rRNA
Prehn-Kristensen et al., 2018 (54)	n/a	Fecal	Shannon	16s rDNA
Wan et al., 2020 (74)	n/a	Fecal	Shannon, Simpson, Chao1	shotgun metagenomic sequencing
Wang et al., 2020 (70)	Actinobacteria, Bacteroidetes, Firmicutes	Fecal	Shannon, Simpson, Chao1	16s rRNA

rRNA, ribosomal ribonucleic acid; DNA, deoxyribonucleic acid; n/a, not applicable due to not being reported.

The most frequently used indices comparing microbiome samples were the OTUS, Chao1, Shannon diversity, and Simpson measures (**Table 4**). Each study provided different classifications and types of bacteria, which are represented in **Table 4**. The OTU and alpha diversity indices of bacterial phyla were used in the present meta-analysis to minimize variation due to disparate levels of classification specificity across studies. The three commonly cited phyla included in the present meta-analysis were Actinobacteria, Bacteroidetes, and Firmicutes (60, 70).

### 3.5 Statistical Meta-Analysis

Both fixed and random-effects models were used to assess estimation error within and between studies. Heterogeneity across studies was assessed using the *I^2^* statistic, with higher values indicating greater percentages of variation across studies that are not attributed to chance (78). A random-effects model was utilized for studies with above midpoint heterogeneity levels (*I^2^* > 50%). In all other cases, a fixed-effect model was adapted (78). The standardized mean difference (SMD) statistic was used to report on continuous variables: an SMD greater than 0 represents the children with ADHD as having greater bacterial diversity or heart rate reactivity, and an SMD less than 0 represents children with ADHD as having lower bacterial diversity and heart rate reactivity (78).

#### 3.5.1 Task-Related Heart Rate Reactivity

##### 3.5.1.1 All Tasks

The pooled estimate of all five included studies showed an insignificant fixed-effect SMD of 0.08 (*p* = 0.27; 95% CI: -.06 to.22) and between-study heterogeneity (*I^2^*) of 66.31% for the HRR differences between children with ADHD and without ADHD (1, 14, 45, 47, 73). These results are represented in **Supplementary Figure 1A**.

##### 3.5.1.2 Cognitive Tasks

Two studies utilized cognitive tasks as an experimental stressor. One study utilized the computerized Tower of London planning task, which requires executive functioning skills to plan, memorize, and manipulate computerized blocks across four difficulty levels and construct a tower (73). Another study utilized the Continuous Performance Test (CPT), an attention stressor (1). A fixed-effects meta-analysis showed a pooled SMD estimate of -0.04 (*p* = 0.68; 95% CI: -0.211 to 0.14) and low heterogeneity (*I^2^* = 0.92%), as shown in **Supplementary Figure 1B**.

##### 3.5.1.3 Emotional Tasks

Two studies used the same emotional task, the International Affective Picture System (IAPS), as the psychological stressor (45, 47). A fixed-effects meta-analysis revealed a pooled SMD estimate of -0.23 (*p* = .21; 95% CI: -0.58 to 0.13). A low heterogeneity between studies was observed (*I^2^* = 34.96%). Data are displayed in **Supplementary Figure 1C**.

#### 3.5.2 Gut Microbiota

##### 3.5.2.1 Operational Taxonomic Unit (OTU)

The differences in gut bacteria OTU were analyzed from three studies and are represented in **Supplementary Figure 2** (54, 60, 74). The OTUs in children with ADHD are not significantly different from the controls (95% CI: -0.06 to 0.48; *p* = 0.83), with a pooled random-effects SMD estimate of -0.06 and no between-study heterogeneity (*I* ^2^ = 0%).

##### 3.5.2.2 Shannon Index

All five studies assessing gut microbiota reported bacterial diversity between the ADHD and control groups using the Shannon index (54, 60, 70, 71, 74). A fixed-effect meta-analysis showed moderate heterogeneity levels between studies (*I^2^* = 60.76%). The pooled fixed-effects SMD estimate was 0.08 between the two groups (*p* = 0.50) and 95% CI between -0.16 and 0.32. A random-effects model revealed a pooled SMD of 0.06 (95% CI: *p* = 0.78) and low heterogeneity attributed to chance across studies (*I^2^* = 15.27%). The 95% CI for the random-effects model was from -0.34 to 0.47 (**Supplementary Figure 3**).

##### 3.5.2.3 Simpson Index

Three studies utilized the Simpson index (70, 71, 74). Heterogeneity was low between studies (*I* ^2^ = 27.75%). The fixed-effects pooled SMD estimate was 0.21 (*p* = 0.18) with a 95% CI of -0.10 to 0.51 (**Supplementary Figure 4**).

##### 3.5.2.4 Chao1 Index

**Figure 3:**
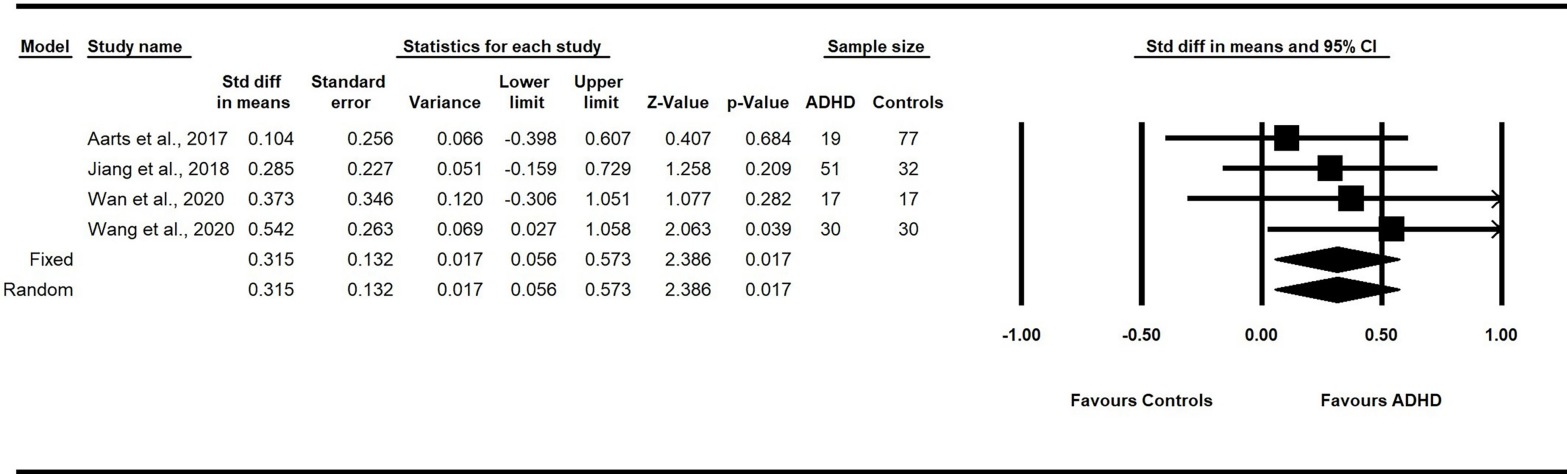
Forest plot of the pooled effect sizes in the standardized difference in means for the Chao1 index in the fecal samples of children with and without ADHD across all studies. Children with ADHD have greater alpha diversity, as measured by the Chao1 index, compared to healthy children without ADHD (*p*<.05). *ADHD*, Attention-Deficit/Hyperactivity Disorder; *CI*, confidence intervals.

Four studies reported bacterial diversity utilizing the Chao1 index (60, 70, 71, 74). There was no evidence of between-study heterogeneity due to chance (*I* ^2^ = 0%). A fixed-effect model revealed a significant pooled SMD estimate of 0.32 with a 95% CI from 0.06 to 0.6 (p = 0.02). These findings suggest that children with ADHD have greater alpha diversity, as measured by the Chao1 index than healthy children without ADHD (**Figure 3**).

##### 3.5.2.5 Actinobacteria

**Figure 4:**
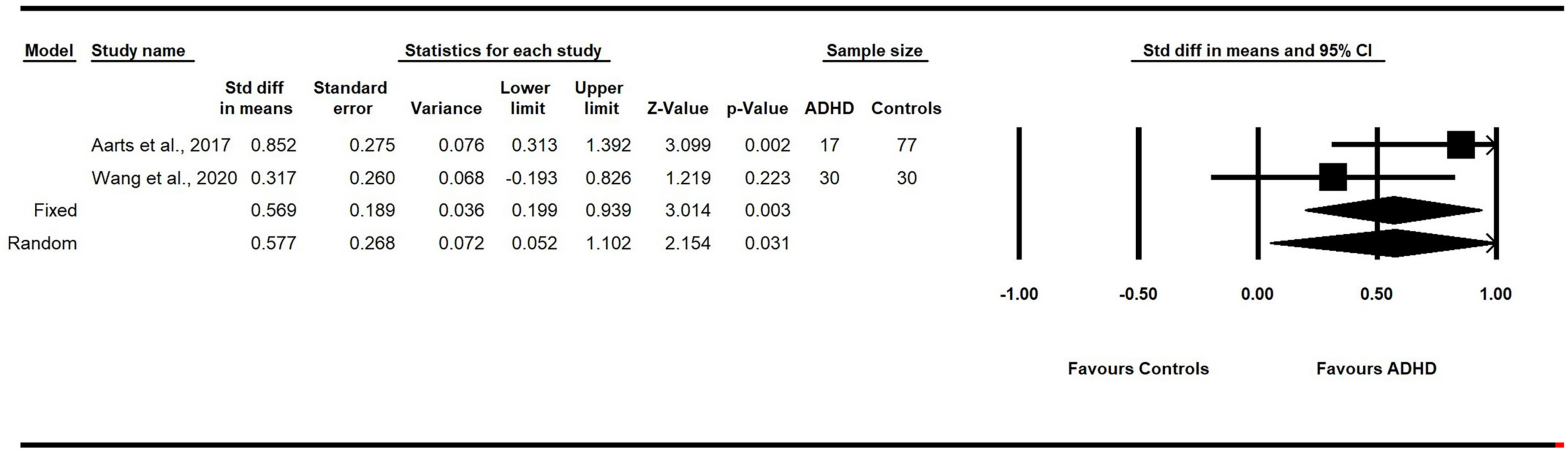
Forest plot of the pooled effect sizes in the standardized difference in means for the Actinobacteria in the fecal samples of children with and without ADHD across all studies. Children with ADHD have significantly greater concentrations of Actinobacteria in their fecal samples than healthy controls without ADHD (*p*<.05). ADHD, Attention-Deficit/Hyperactivity Disorder; CI, confidence intervals.

Actinobacteria are a phylum of anaerobic bacteria, consisting mostly of *Bifidobacteria*, associated with vaginal delivery and breastfeeding during the perinatal and postnatal stages, maintaining homeostasis, and controlling inflammation (79). Two studies reported the relative abundance of Actinobacteria between groups (60, 70). A fixed-effects meta-analysis showed a pooled SMD estimate of 0.57 (*p* < 0.01; 95% CI: 0.20 to 0.94) and moderate heterogeneity (*I^2^* = 50.13%). A random-effects model revealed a pooled SMD estimate of 0.58 (*p* = .03, 95% CI: 0.05 to 1.1) and no between-study heterogeneity (*I^2^* = 0%). These findings suggest that children with ADHD have significantly greater concentrations of Actinobacteria in their fecal samples than healthy controls without ADHD (**Figure 4**).

##### 3.5.2.6 Bacteroidetes

**Figure 5:**
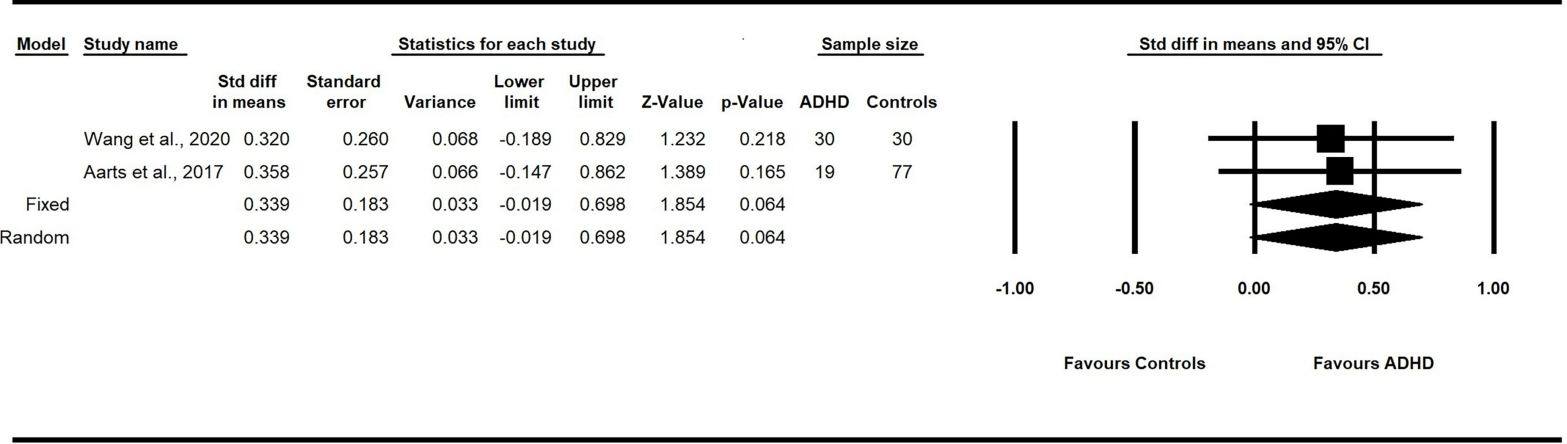
Forest plot of the pooled effect sizes in the standardized difference in means for the Bacteroidetes in the fecal samples of children with and without ADHD across all studies. Bacteroidetes may be elevated in children with ADHD compared to healthy controls without ADHD (*p*=.064). ADHD, Attention-Deficit/Hyperactivity Disorder; CI, confidence intervals.

The Bacteroidetes phylum comprises a diverse group of bacteria that aid in metabolizing complex carbohydrates for energy retrieval (80). Two studies reported the relative abundance of Bacteroidetes in the fecal samples of children with and without ADHD (60, 70). A fixed-effect model yielded a pooled SMD estimate of 0.34 (p = .06; 95% CI: -0.02 to 0.70) and no heterogeneity due to chance between studies (*I* ^2^ = 0%). The concentration of Bacteroidetes between children with and without ADHD did not reach statistical significance (**Figure 5**).

##### 3.5.2.7 Firmicutes

**Figure 6:**
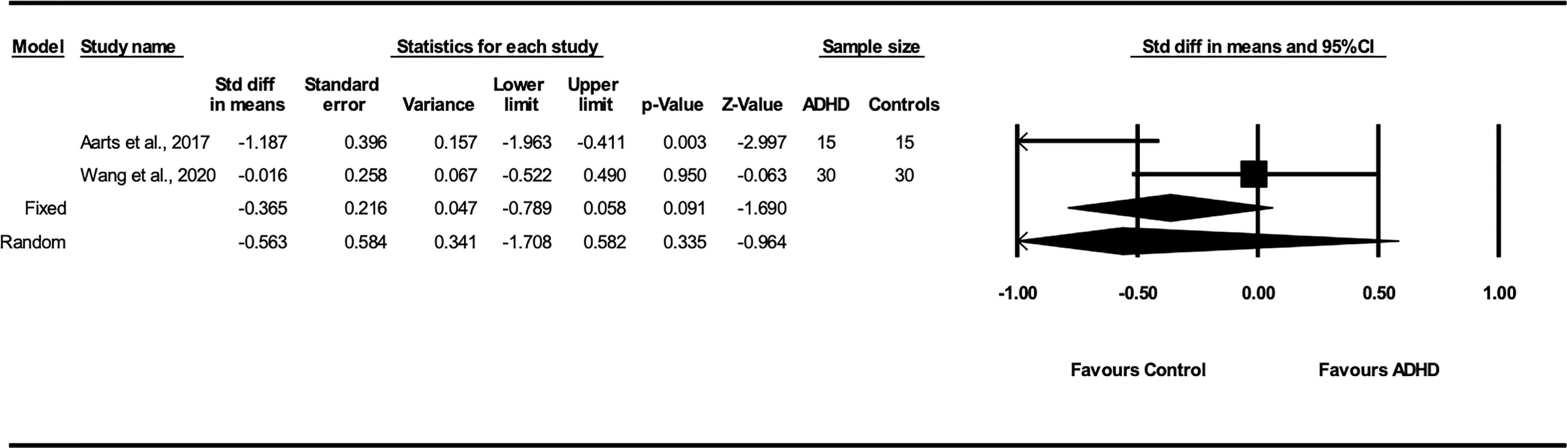
Forest plot of the pooled effect sizes in the standardized difference in means for the Firmicutes in the fecal samples of children with and without ADHD across all studies. Firmicutes may be lower in children with ADHD compared to healthy controls without ADHD (*p*=.091). *ADHD*, Attention-Deficit/Hyperactivity Disorder; *CI*, confidence intervals.

Two studies reported the relative abundance of the Firmicutes phylum in the fecal samples of children with and without ADHD (60, 70). A fixed-effects model revealed an estimated SMD of -0.37 (p = 0.09; 95% CI: -1.71 to 0.58) and had high between-study heterogeneity (*I* ^2^ = 83.69%). The random-effects model showed a non-significant pooled SMD estimate of -0.56 (p = 0.33; 95% CI: -1.71 to 0.58). These findings suggest that the concentration of Firmicutes in the gut microbiome of children with and without ADHD was not statistically different (**Figure 6**).

## 4 Discussion

The present meta-analysis sought to quantify the strength of the association between peripheral physiological functioning, task-related HRR or gut microbiota composition, and the diagnosis of childhood ADHD. Given that some core ADHD symptoms (i.e., impulsivity and hyperactivity) are analogous with poor emotional regulation (43), examining the possible physiological underpinnings of emotional dysregulation can improve our understanding of the pathogenesis of ADHD and provide additional mechanisms for treatment. The physiological link between emotional dysregulation and ADHD reflects interconnected but distinct, psychological, neurological, and genetic etiological and developmental processes (44). To date, no study reviewed by the authors has examined the effect sizes of task-related heart rate reactivity and gut physiological markers within the context of childhood ADHD. We conducted a meta-analytic review of available evidence to evaluate the difference in task-related HRR or gut microbiota in children with ADHD and their non-ADHD peers published within the past decade.

Although prior work suggested differences in microbiota populations in children with ADHD (59, 68, 75) and provided some evidence for altered ANS by measuring HRV in children with ADHD (40), this is believed to be the first effort to examine task-related HRR and microbiota alterations in children with ADHD. We found evidence for altered gut microbiota in children with ADHD but no conclusive evidence for task-related HRR differences. These findings partially support prior research regarding physiological evidence for emotional dysregulation in ADHD (81, 82).

### 4.1 Task-Related Heart Rate Reactivity in Children With ADHD

CVC, measured *via* task-related HRR, was assessed in the five case-control studies that met the criteria for inclusion (1, 14, 45, 47, 73). Contrary to expectations, no significant patterns emerged between the ADHD group and the controls. In addition, further analyses by task type assessing the effect size from studies that employed either a cognitive or emotional task did not find significant evidence of altered task-related HRR in the ADHD group. However, given that this research area is fairly underdeveloped, any non-significant effect sizes reported here should not be used to rule out altered autonomic reactivity among youth with ADHD.

#### 4.1.1 Strengths and Weaknesses of the Task-Related Heart Rate Reactivity Studies

There are several limitations to examining differences in altered CVC after a task demand in youth with ADHD. First, many included studies have small sample sizes and high levels of between-study heterogeneity (28). Only two studies reported having enough participants to meet the threshold for statistical power to detect a small to moderate effect (1, 14). The limited number of studies included in this meta-analysis and their small sample sizes thus represent a notable limitation. Second, while some studies in this area employed tasks to elicit arousal or stress that may not generalize to real-world settings, multiple efforts have found differences in HRV by task type, with physical activity tasks having the most prominent effect (28, 83). As such, research in this area would be enhanced by incorporating task types that better align with stressors these children and youth may face, from learning new and challenging academic content to navigating a difficult circumstance with a peer or adult. Third, additional research is necessary to rule out medication and respiration rate as potential confounds (84, 85). The samples included in this meta-analysis ranged from drug-naïve (45) to free of stimulant medication for at least 24 hours before the study (14). No participants were medicated at the time of data collection. Given the evidence that psychostimulants can alter cardiovascular functioning (e.g., 86), researchers should adopt uniform guidelines for stimulant use to strengthen empirical methods and contextualize findings.

### 4.2 Alterations of the Gut Microbiota in Children With ADHD

The present study found significant between-group differences in intestinal microbial diversity across the five included studies. Specifically, the pooled small effect size of Chao1 levels in the gut profile was significantly larger for children with ADHD (60, 70, 71, 74). Follow-up analyses of the microbiota composition at the phylum level revealed that children with ADHD had a medium effect size of significantly greater concentrations of Actinobacteria. These findings support our hypothesis of altered gut microbiota in children and youth with ADHD.

Within the phylum Actinobacteria, the genus *Bifidobacterium* is essential during early development and is expected to taper off during childhood (60). To that end, Partty et al. (87) found that infants who later developed ADHD had *Bifidobacterium* deficiencies and noted the role of *Bifidobacterium* during the breastfeeding stages in mitigating the onset of later childhood neurodevelopmental disorders. Some researchers have observed that an increase of *Bifidobacterium* in the ADHD cohort occurred at the expense of more developmentally appropriate bacteria (66). Perhaps the inverse trajectory of *Bifidobacterium* —that is, its notable deficiency early in life and its dominance during childhood in the gut of children with ADHD— may be an important marker for assessing ADHD risk during infancy (59, 60, 70). While the specific pathway still warrants elucidation, the Actinobacteria phylum appears relevant to childhood ADHD.

Although the differences in concentrations of Bacteroidetes and Firmicutes did not reach significance, results identified trend-level differences (i.e., *p* <.10). The small number of studies investigating these phyla in children with ADHD may have limited the power and sensitivity of the present analysis to detect a significant small effect size. Nevertheless, control groups had greater absolute Firmicutes levels than those with ADHD in both studies reporting these bacterial concentrations (60, 70). While the differences in Firmicutes were not of a magnitude to reach traditional significance levels in each of the two studies, the shared trend of decreased Firmicutes among the ADHD group warrants future study.

In contrast, Bacteroidetes concentrations were lower in the ADHD group in the Wang et al. (70) study but higher in Aarts et al. (60). Because Bacteroidetes are connected to complex carbohydrate consumption, these mixed findings may point to the influence of diet and socioeconomic and cultural variations in diet. Future research targeting Bacteroidetes and Firmicutes composition and their ratio (88) may help address these inconsistent findings. Furthermore, assessing how a broader range of relevant sociocultural and genetic factors may influence possible physiological pathways associated with ADHD or other emotional dysregulation-related disorders may clarify the nature of these associations.

In sum, the results of this meta-analysis found evidence of altered microbiota in children with ADHD. Of note, the number of different bacteria in children with ADHD was higher than the control groups, which was an unexpected finding as greater diversity often indicates better functioning and health (76, 89). However, this perspective stems from adult populations; thus, this finding in children and adolescents requires further investigation. Second, Actinobacteria, consisting mainly of *Bifidobacterium*, were significantly elevated in children with ADHD. We found a medium effect size, suggesting reliable differences within this phylum could be a physiological indicator of ADHD.

Recently, differences in Actinobacteria were reported in a meta-analysis examining the gut microbiota of children with ASD, a neurodevelopmental disorder often comorbid with ADHD (75). The authors found a significant effect size for Bifidobacterium differences; however, children with ASD had lower *Bifidobacterium* differences such that *Bifidobacterium* levels were lower in children with ASD than their neurotypical peers (78). This finding contrasts with the present meta-analysis’ finding of elevated *Bifidobacterium* in children with ADHD (75, 78). Thus, it may be that higher levels of *Bifidobacterium* could serve as a sensitive indicator of ADHD. While further research is necessary to support that possibility, the nature of the present findings suggests that assessing the overall diversity of the fecal microbiota is insufficient for ascertaining ADHD-related patterns; examining the microbiota subtypes making up the gut profile is likely necessary (52).

#### 4.2.1 Strengths and Weaknesses of the Gut Microbiota Studies

While these findings contribute to an emerging literature, it may be premature to draw conclusions about the relative importance of microbiota in neurodevelopmental disorders. For instance, it is not yet known if assessing the metabolic potential of the gut microbiota in conjunction with their produced metabolites is more integral to our understanding of the metabolic influences on emotional regulation than simply measuring microbiota concentration (55). Although differences in microbial composition exist in this review, we did not examine the associated metabolites or neurotransmitters of the microbiome that regulate stress response. Also, the broad age range further limits these findings as ADHD is a neurodevelopmental disorder, and microbiota populations vary across childhood and adolescence.

Other limitations of existing studies include their small sample sizes, ranging from 14 to 51 youth with ADHD in the present meta-analysis (54, 71). In addition, two studies included only males (14, 54). While these studies were conducted in various countries, thus enhancing generalizability, this also increased the complexity of varying genetic risk and sociocultural factors such as diet composition and use of pre and probiotic supplements (55, 64, 65, 70, 87). Finally, results may have been further influenced by non-standard stool sample preparation methods, nucleic acid extraction, or bioinformatics analysis (66). Thus, future studies in this area should employ more rigorous and consistent methods for tracking medication use, diet, intestinal motility (i.e., stool frequency), and other related sociocultural factors that influence the gut microbiota and ADHD prevalence (55).

### 4.3 Future Directions for Research

Future research into the role of ANS activity in childhood ADHD should employ appropriate task stimuli that can best represent the circumstances and environmental contexts that contribute to externalizing symptoms in children with ADHD. Although many lab-based tasks may be stress-inducing, the nature of the stress reactivity exhibited by children with ADHD at school and home may involve different mechanisms sensitive to HRR. One such way to strengthen the external validity of study designs is to conduct studies with heart rate monitors and collect data across the day *via* ecological momentary assessment (EMA) methodologies (50).

As a heterogenous behavioral disorder, examining the physiological correlates of the three ADHD subtypes might provide critical information for developing tailored treatment plans. These subtypes were not consistently reported in many studies included in this report (47, 60, 71, 73, 74). Although specifying the ADHD subtype may split participants into smaller subsamples and reduce power to detect differences, categorizing may offer helpful clinical information on the differential etiology, trajectory, and treatment of ADHD.

Similarly, the findings of this meta-analysis further highlight the utility of defining ADHD biotypes. Biotyping differentiates ADHD by physiological differences irrespective of phenotypic presentation and has potential clinical and research implications (40). Different interventions have varying effects on physiological functioning; thus, identifying biotypes may enhance our ability to treat individuals with ADHD (40). For example, biofeedback therapy has been linked with decreased hyperactivity symptoms in children with ADHD (90). Specific therapies may have different effects on physiological functioning; thus, biotyping may enhance our ability to tailor ADHD treatments.

Future studies should also contribute to the growing work on differentiating ADHD by tracking information relevant to its diverse comorbidity presentation. This recommendation holds for the multiple conditions that regularly co-occur with ADHD, from other externalizing disorders (e.g., CD, ODD) and internalizing disorders (e.g., mood and anxiety disorders) to learning disabilities and other neurodevelopmental conditions (e.g., ASD; e.g., 91–94). A growing body of literature on the endophenotypes of ADHD suggests that endophenotypes may provide a mechanism for how ADHD differentially develops between siblings (95). Given that siblings may share similar diets and cultures and common genetic compositions, utilizing siblings as a control group for these studies may be an additional avenue of research.

Additionally, it will be important for researchers in this area to establish standardized protocols that permit appropriately nuanced evaluation of the microbiome and its potential contribution to ADHD. Developmental and contextual factors should be considered, given that gut microbiota composition fluctuates throughout the lifespan (40, 59, 76) and optimal gut microbiota for healthy human development are culture- and age-dependent (59). Such contextual factors could include diet composition; notably, only one study in this meta-analysis utilized a validated food frequency questionnaire (70). Finally, the current literature has not established the most efficient taxonomic level to compare gut microbiota—the present study employed two ways to assess gut microbiota: index and taxonomic unit.

## 5 Applications And Future Directions

Subsequent research may inform the application of findings regarding task-related HRR and other ANS indicators to the growing use of yoga, mindfulness, and other meditative interventions in youth with ADHD (e.g., 96). Similarly, task-related HRR data may yield benefits for biofeedback-based strategies. These approaches and others that typically aim to increase self-regulation, inhibitory control, and emotional regulation (97–99) may be more efficacious in treating externalizing rather than internalizing symptoms of ADHD and its comorbid conditions. As such, boys with ADHD may benefit more from this treatment as they often present with more externalizing symptoms than girls with ADHD (100).

Regarding gut microbiota, these findings support prior research that specific dietary components can modify the gut microbiota and alter activity in brain regions relevant to cognition, behavior, and symptoms of ADHD (64, 65, 70). As such, probiotic supplementation may positively affect the course of neurodevelopmental disorders, including ADHD (59, 61, 87). Alterations in diet and probiotic therapy recommendations can be considered potential adjunctive or complementary therapies to traditional treatment modalities (e.g., psychostimulants, psychotherapy). Additionally, screening and dietary monitoring can improve the reliability and validity of these studies by refining the translational clinical utility of the findings.

Finally, the results of this review support using a combination of physiological and psychological measures in laboratory and real-world contexts to assess the relationship between emotional dysregulation and ADHD or the embodiment of emotional dysregulation linked to non-neurotypical development. Developmental stage, diet, gastrointestinal health, and medication use are also pertinent factors to consider. We recommend using behavioral and physiological measures to study the heterogeneous etiology and developmental trajectory of ADHD in children and adolescents to distinguish biotypes by stress response patterns and behavioral profiles.

Physiotherapy methods, such as yoga, biofeedback breath control training, and probiotic biotherapy, can be paired with psychotherapy to help address physiological self-regulation (90). Evidence-based nonpharmacological treatments include parent training, school-based interventions, multicomponent summer camp models, and social skills training, which have the added benefit of creating supportive and collaborative environments between the youth’s school, family, and more (e.g., 2, 101). Combining these nonpharmacological treatments and the cascading effects on the heart and brain will shed light on the pathways and processes that contribute to ADHD and its varying presentations and guide the use of well-targeted multicomponent interventions.

## 6 Conclusion

Existing evidence underscores that ADHD can no longer be conceptualized as primarily a neurocognitive disorder driven by a neurotransmitter imbalance. Due to impulsivity and hyperactivity symptoms, emotional dysregulation has also gained traction as a key component of ADHD (43). Given that the developing child embodies a complex and adaptive human system influenced by multiple biopsychosocial factors, more robust observational, cohort, and case studies using the whole-body approach to examine the physiological associations of emotional dysregulation in ADHD are needed.

Although limited by the small number of studies included in the present meta-analysis, our findings suggest that gut microbiota disruptions are linked to childhood ADHD; whether changes in the gut microbiota are a causal factor or symptom of the disorder remains unknown. However, these results suggest that utilizing a whole person lens – multiple physiological systems existing within several environmental contextual layers (102, 103) – will be necessary to enhance understanding of the development and maintenance of childhood ADHD. Additionally, the current results may have implications for expanding or refining clinical assessments and interventions with patients with ADHD. In conclusion, by looking beyond the brain, peripheral physiological markers may offer insights into how early childhood development may be modified and linked to neurodevelopmental disorders, equipping researchers, clinicians, and educators to better assist the increasing global population living with ADHD.

## Data Availability Statement

The original contributions presented in the study are included in the article/**Supplementary Material**. Further inquiries can be directed to the corresponding author.

## Author Contributions

As lead author, AP conceived the study aims, led the writing project, completed data analyses, created tables and figures, and was the primary writer for the majority of the first manuscript draft. MJC, TGC, and JMB were primary writers for portions of the introduction and discussion. AP, MJC, TGC, and JMB were involved in conducting the publication search and review of papers for meeting inclusion criteria. RPK provided clinical expertise and critical feedback during the writing and editing process. As senior author, JMB provided financial support for the statistical software and publication costs as well as oversaw and guided the study from inception through dissemination. All authors have edited, reviewed and approved the final version of the manuscript. 

## Funding

JMB secured funds to support the analysis and publication of this paper; internal funds were awarded by the UNC Charlotte’s Atkins Library and Department of Psychological Science for publishing an open access article. In addition, JMB is supported by research funds from earning the Bonnie E. Cone Early-Career Professorship in Teaching. AP’s time and effort were supported by funds from the UNC Charlotte SciComm Fellowship grant from Burroughs Wellcome Fund, Psi Chi APAGS Junior Scientist Fellowship, UNC Charlotte Loch Walker Writing Award, the MENSA McGrew-Fruecht Scholarship, and the Health Psychology PhD program.

## Conflict of Interest

The authors declare that the research was conducted in the absence of any commercial or financial relationships that could be construed as a potential conflict of interest.

## Publisher’s Note

All claims expressed in this article are solely those of the authors and do not necessarily represent those of their affiliated organizations, or those of the publisher, the editors and the reviewers. Any product that may be evaluated in this article, or claim that may be made by its manufacturer, is not guaranteed or endorsed by the publisher.
