# Supplementary material for: Childhood ADHD, Going Beyond the Brain: A Meta-Analysis on Peripheral Physiological Markers of the Heart and the Gut
Source: Front Endocrinol (Lausanne). 2022 Mar 1;13:738065. doi: 10.3389/fendo.2022.738065 (PMC8921263; doi:10.3389/fendo.2022.738065)
Supplement: Supplementary file 1 [file DataSheet_1.pdf]

Model	Study name	Std diff in means	Standard error	Variance	Lower limit	Upper limit	Z-Value	p-Value	ADHD	HC
		Statistics for each study								
	Dekkers et al., 2020	0.442	0.152	0.023	0.145	0.739	2.915	0.004	81	99
	Griffiths et al., 2017	-0.007	0.094	0.009	-0.191	0.177	-0.075	0.940	224	229
	Perrin et al., 2014	0.356	0.289	0.083	-0.210	0.921	1.233	0.218	19	34
	Souroulla et al., 2020	-0.444	0.253	0.064	-0.939	0.051	-1.757	0.079	24	48
	Taskiran et al., 2017	-0.003	0.266	0.071	-0.525	0.518	-0.013	0.990	48	20
Fixed		0.079	0.071	0.005	-0.060	0.218	1.114	0.265		
Random		0.082	0.146	0.021	-0.204	0.369	0.564	0.572		

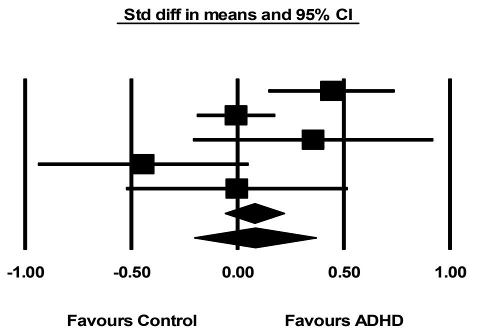

A.

Model	Study name	Std diff in means	Standard error	Variance	Lower limit	Upper limit	Z-Value	p-Value	ADHD	Controls
		Statistics for each study							Sample size	
	Griffiths et al., 2017	-0.007	0.094	0.009	-0.191	0.177	-0.075	0.940	229	224
	Perrin et al., 2014	-0.311	0.288	0.083	-0.876	0.253	-1.081	0.280	19	34
Fixed		-0.036	0.089	0.008	-0.211	0.139	-0.407	0.684		
Random		-0.037	0.091	0.008	-0.216	0.141	-0.411	0.681		

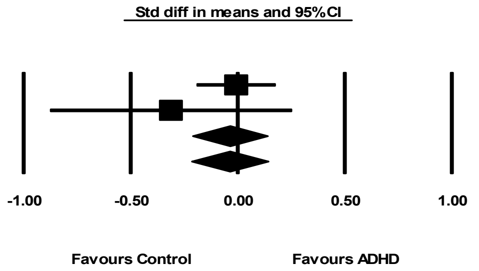

B.

Model	Study name	Std diff in means	Standard error	Variance	Lower limit	Upper limit	Z-Value	p-Value	ADHD	Controls
		Statistics for each study							Sample size	
	Souroulla et al., 2019	-0.444	0.253	0.064	-0.939	0.051	-1.757	0.079	24	48
	Taskiran et al,. 2017	0.003	0.257	0.066	-0.501	0.508	0.013	0.990	48	22
Fixed		-0.225	0.180	0.033	-0.578	0.129	-1.245	0.213		
Random		-0.223	0.224	0.050	-0.662	0.215	-0.998	0.318		

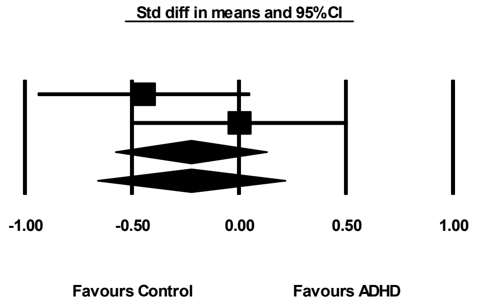

C.

Supplementary Figure 1. Summary of the task-related heart rate reactivity analyses. Forest plot of the pooled estimate of heart rate reactivity standardized mean differences between children with and without ADHD across (A) all tasks, (B) cognitive tasks, and (C) emotional tasks. All analyses were non-significant (p's >.05). *ADHD* = ADHD: Attention-Deficit/Hyperactivity Disorder; *HC* = health controls; *CI* = confidence intervals.

Model	Study name	Statistics for each study							Sample size	
		Std diff in means	Standard error	Variance	Lower limit	Upper limit	Z-Value	p-Value	ADHD	Control
	Aarts et al., 2017	-0.270	0.257	0.066	-0.773	0.234	-1.051	0.293	19	77
	Wang et al., 2020	0.437	0.261	0.068	-0.075	0.949	1.672	0.094	30	30
	Prehn-Kristensen et al., 217	-0.424	0.365	0.133	-1.139	0.292	-1.161	0.246	14	17
Fixed		-0.023	0.164	0.027	-0.344	0.298	-0.143	0.887		
Random		-0.057	0.271	0.073	-0.588	0.474	-0.210	0.834		

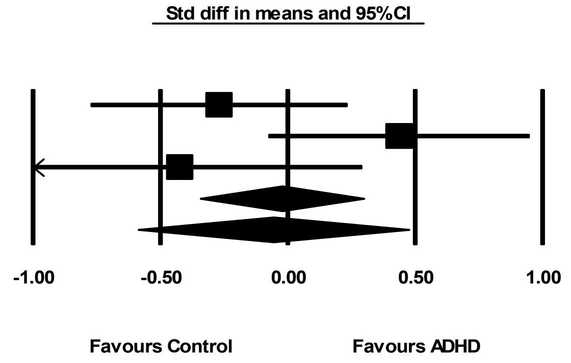

Supplementary Figure 2. Forest plot of the pooled effect sizes in the standardized difference in means of operational taxonomic unit (OTU) in the fecal samples of children with and without ADHD across all studies. Analysis was non-significant (*p* >.05). ADHD: Attention-Deficit/Hyperactivity Disorder; *CI* = confidence intervals.

Model	Study name	Statistics for each study							Sample size	
		Std diff in means	Standard error	Variance	Lower limit	Upper limit	Z-Value	p-Value	ADHD	Control
	Aarts et al., 2017	-0.107	0.256	0.066	-0.609	0.396	-0.417	0.677	19	77
	Jiang et al., 2021	0.057	0.226	0.051	-0.385	0.499	0.254	0.799	51	32
	Prehn-Kristensen et al., 2017	-0.794	0.375	0.140	-1.528	-0.059	-2.118	0.034	14	17
	Wan et al., 2020	0.434	0.347	0.120	-0.246	1.114	1.251	0.211	17	17
	Wang et al., 2020	0.549	0.263	0.069	0.033	1.064	2.087	0.037	30	30
Fixed		0.083	0.124	0.015	-0.160	0.327	0.669	0.503		
Random		0.057	0.204	0.042	-0.342	0.457	0.282	0.778		

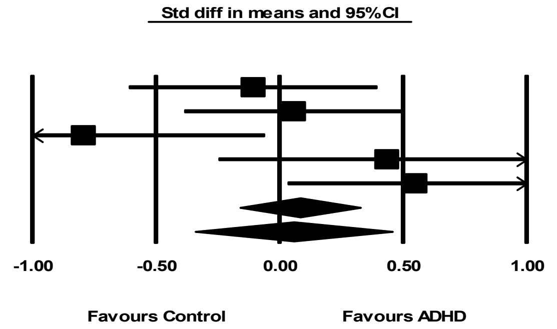

Supplementary Figure 3. Forest plot of the pooled effect sizes in the standardized difference in means of Shannon index in the fecal samples of children with and without ADHD across all studies. Analysis was non-significant (*p* >.05). ADHD: Attention-Deficit/Hyperactivity Disorder; *CI* = confidence intervals.

Model	Study name	Statistics for each study								
		Std diff in means	Standard error	Variance	Lower limit	Upper limit	Z-Value	p-Value	ADHD	HC
	Jiang et al., 2018	-0.025	0.226	0.051	-0.467	0.417	-0.109	0.913	51	32
	Wang et al., 2020	0.549	0.263	0.069	0.033	1.064	2.087	0.037	30	30
	Wan et al., 2020	0.153	0.344	0.118	-0.520	0.827	0.447	0.655	17	17
Fixed		0.205	0.153	0.023	-0.095	0.506	1.341	0.180		
Random		0.214	0.184	0.034	-0.146	0.574	1.167	0.243		

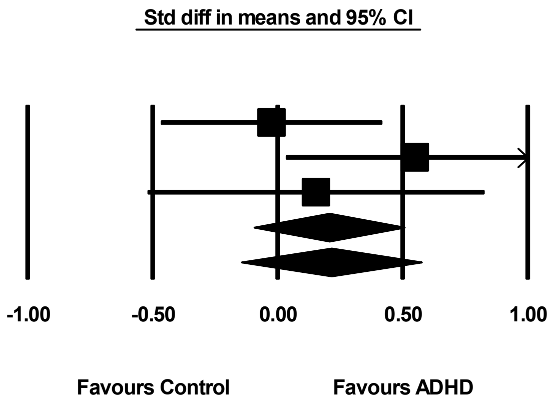

Supplementary Figure 4. Forest plot of the pooled effect sizes in the standardized difference in means of Simpson index in the fecal samples of children with and without ADHD. Analysis was non-significant (p >.05). *ADHD* = ADHD: Attention-Deficit/Hyperactivity Disorder; *HC* = health controls; *CI* = confidence intervals.
